# Supplementary material for: GPMelt: A hierarchical Gaussian process framework to explore the dark meltome of thermal proteome profiling experiments
Source: PLoS Comput Biol. 2024 Sep 27;20(9):e1011632. doi: 10.1371/journal.pcbi.1011632 (PMC11463780; doi:10.1371/journal.pcbi.1011632)
Supplement: Attachment — Submitted filename: GPMelt_ResponseToReviewers.pdf [file pcbi.1011632.s002.pdf]

Hierarchical Gaussian process models explore the dark meltome of thermal proteome profiling experiments.

Cecile Le Sueur[1], Magnus Rattray[2☯*], Mikhail Savitski[1☯*]

1 Genome Biology Unit, European Molecular Biology Laboratory, 69117 Heidelberg, Germany
2 Faculty of Biology, Medicine and Health, University of Manchester, Manchester M13 9PT, UK

☯These authors jointly supervised this work.
* magnus.rattray@manchester.ac.uk, mikhail.savitski@embl.de

General changes in notation

Following the Guest Editor and reviewers comments, we decided to update some notation to improve the text clarity. We hereafter list these changes:

- In the original manuscript, we used the denomination "alternative" models to refer to the hierarchical Gaussian process (HGP) models $\mathcal{M}_1$ for which all conditions are modelled as a different task in the multi-task framework. We believe that these models, **fully** describing each condition in isolation as a task, would be better denoted by "full models".

- Similarly, we propose to update the denomination "null" model. In the manuscript, a null model $\mathcal{M}_0$ corresponds to a model in which at least two conditions are **jointly** modelled as one task, hence we replaced "null" by "joint" models. We hope that this could be beneficial for the reader to better understand that, in presence of multiple conditions, multiple "joint" models could be defined, depending on which conditions are actually "jointly" modelled.

- We changed the symbol of our statistic from LR to Λ. Indeed, we realised that LR, standing for *likelihood ratio*, was a confusing name. Our statistic is **related but different** from a standard likelihood ratio test statistic, and therefore should not be confused with a likelihood ratio test statistic whose properties are fairly well-known and studied.

- In the original manuscript, we denoted by θ_p the type II Maximum Likelihood Estimation (MLE) of the HGP model parameters. We realised that this notation might be confusing, as there is no way to distinguish the set of *unknown* (unestimated) parameters to the set of *estimated* ones. Therefore, we updated the notation such that we denote the type II MLE of the HGP model parameters by θ_p^{MLE-II}, and by θ_p the unknown HGP model parameters.

- Finally, we replaced ABC_{HGPM} by ABC_{GPMelt}. ABC_{HGPM} stood for "Area Between the Curves computed from the predictive distribution obtained by fitting the HGP model to the data". We believe that directly linking this ABC computation to the presented method, GPMelt, will make it easier to the reader to distinguish this ABC computation from ABC_{median}, the ABC approximation using the median observations.

Guest Editor comments to authors

Comment 1

The manuscript clearly demonstrate mastery of some challenging statistical concepts, however, given the denseness of the mathematical components of the model it would be helpful to increase the accesibility of the text to those who may use the model rather than fully comprehend all the details. I suggest the authors include a high-level description of the method early on the methods sections and a summary of the main algorithmic steps at the end of the methods section.

We thank the guest editor for this suggestion, and updated Figure 1 (p.4 of the main text) from the main to provide a high-level description of the method. The main algorithmic steps have also been represented schematically and added as Supplementary Figure S1 (p.30 of the supplement text) for space reasons. The following changes have been made to the text:

- Legend of Fig 1 has been updated as follows: "(A) Temperature-Range Thermal Proteome Profiling (TPP-TR) protocol: 10 aliquots of cells or cell lysates are heated to a range of temperatures for three minutes. Subsequently the formed aggregates are removed by filtration or centrifugation. The soluble fractions is digested with trypsin and labeled with tandem mass tags and then analyzed by mass spectrometry. Acquired data, which are on the peptide-level, can be combined to obtain the protein-level melting curves, by averaging over the measurements of all tryptic peptides corresponding to a single protein entry in the protein database. (B) The GPMelt framework consists in fitting simultaneously all replicates of all conditions independently for each protein, using a hierarchical Gaussian process (HGP) model (1). This model, denoted by $\mathcal{M}_1$, is called the *full* model. The full model is fitted via type II maximum likelihood estimation (type II MLE), and estimated parameters θ_p^{MLE-II} are plugged into the so-called *joint* models (3). A joint model $\mathcal{M}_0$ corresponds to a model in which at least two conditions are jointly modeled as one (represented in grey in $\mathcal{M}_{0,Cd1}$ and $\mathcal{M}_{0,Cd2}$). The joint models are used to generate an approximation of the null distribution of the GPMelt statistic Λ (4). The observed statistics $\Lambda_{Ctrl\ vs\ Cd1}$ and $\Lambda_{Ctrl\ vs\ Cd2}$ (2) are compared to this null distribution approximation to compute empirical p-values (5). Additionally, an effect size is computed using the predicted fits obtained from $\mathcal{M}_1$ (6)."

- We updated l.71-72 (p.3 of the main text) by: "A high-level visualisation the TPP-TR protocol and GPMelt framework is depicted in Fig 1."

- The following sentence has been added after l.81 (p.3 of the main text): "A summary of the main algorithmic steps is represented in S1 Fig".

Comment 2

The advancements over ref. [1] are evident but it is not clear that this prior framework doesn't already overcome the sigmoid assumption. Appreciating the computational cost is perhaps prohibitive, it may be worthwhile demonstrating the differences empirically on a few select examples?

We apologize if it appeared to the Guest Editor that the manuscript is not clear enough about the fact that Fang et al. [1] already overcomes the sigmoid assumption. We fully acknowledge that Fang et al. [1] pioneered the use of Gaussian processes to include non-sigmoidal melting curves in TPP analysis. In the present work, we build on [1] to develop a hierarchical model able to incorporate multiple conditions. Additionally, the HGP model is shown to be more robust to outlier observations and replicates (see Fig 6 panels A and B, p.32 of the main text).

The following sentences in the manuscript were meant to emphasize that Fang et al. [1] first introduced Gaussian processes to overcome the sigmoidal assumption:

- l.153-163 (p.6-7 of the main text): "However, the main limitation of NPARC remains the sigmoidal assumption.[...] To deal with this limitation, Fang et al.[17] proposed an updated version of their Bayesian sigmoid model, called the Bayesian semi-parametric model, in which an additional term, a zero-centered GP, captures correlated residuals and model deviations from the sigmoidal assumption."

- l.443-444 (p.15 of the main text): "Having shown that GPMelt performs better than the Bayesian semi-parametric model while being based on a similar principle of non-sigmoidal curves inclusion, [...]"

- l.766-768 (p.21-22 of the main text): "Pioneering the use of Gaussian processes to incorporate non-sigmoidal melting curves in TPP analysis, Fang et al.[17] developed a so-called Bayesian semi-parametric model."

The following sentence "Our model is conceptually in line with the Bayesian semi-parametric model[17], but we suggest to fully relax the sigmoidal assumption and to set $m \equiv 0$. " (l.169-172 , p.7 of the main text) has been changed to:

- "Fang et al.[17] allowed for non-sigmoidal curves by adding a GP to the sigmoidal mean function. We relax the sigmoidal assumption by setting the mean to zero ($m \equiv 0$) and instead use correlations across replicates and conditions to capture typical behaviours through hierarchical modelling."

Comment 3

It is unclear whether the p-values arising from the authors model are well-calibrated, since the authors make some remarks about being careful with p-value distribution it would be good to show empirical FDR versus observed FDR for some example especially as the hierarchy gets deeper. If the authors are struggling for a while defined ground truth it may help to generate known differences of different AUC sizes and resample residuals according to an appropriate null and alternative models. It would be useful to for user to understand how much they can trust the p-value as a measure of uncertainty quantification in this way.
We thank the Guest Editor for this important comment, and demonstrate using synthetically generated data that GPMelt's statistic Λ leads to well calibrated p-values with appropriate control of the FDR. For the sake of the demonstration, we generated synthetic data according to GPMelt full and joint models, for hierarchical models with three or four levels, and with two or three conditions. We also varied the amount of correlated noise induced in our synthetic data, going up to 1000 times more than observed in clean real data. We do not expect observations of all proteins in a TPP-TR dataset to be 1000 times noisier than the clean baseline data we used hereafter, but this could happen for few proteins, hence we consider this scenario to test the performance of GPMelt in extreme conditions.

Synthetic datasets generation While it is relatively easy to simulate sigmoidal melting curves with known differences in AUC, it is more complicated to predict differences in AUC for non-sigmoidal data, like the ones GPMelt has been developed to deal with. What can be done instead is to define appropriate parameters for the full hierarchical GP model $\mathcal{M}_1$ (i.e. the model in which each condition is modeled as a separate task in a multi-task framework) and sample from it. If the parameters are correctly defined, obtained samples will typically present systematic differences between conditions, hence providing a way to simulated *true differential* observations. Importantly, using similarly defined parameters together with the joint hierarchical GP model (i.e. the model in which at least two conditions are modeled as a shared task), one can generate *true negative* observations.

To define appropriate parameters, we selected 160 protein IDs from the ATP 2019 dataset [2] for which the GPMelt statistic Λ was found to be among the 200 largest ones, and for which the computed absolute ABC from GPMelt fit is among the top 80%. This selection ensures that the real observations for these proteins are really clean (i.e. reproducible replicates inside a condition and large enough difference between conditions). Using these proteins, we considered the associated estimated type II MLE parameters θ^{MLE-II} as basis to generate synthetic data.

We hereafter describe the general algorithm used to generate synthetic data from a hierarchical GP model with L levels, C conditions and R replicates in total (with

$R_c = \frac{R}{C}$ a constant number of replicates per condition). Given such a model, the likelihood of the observations for one protein p can be expressed as:

$$Y_p | T_p, \theta_p \sim \mathcal{N}(\mathbf{0}, \Sigma_p(T_p, T_p) + \beta_p^2 I_{N_p}) \ . \tag{1}$$

Getting rid of the index p for simplicity, we propose to rewrite Σ in terms of index kernels and correlation matrix as follows:

$$\Sigma = \left(\sum_{l=1}^{L-1} K^l \right) \circ K^{t,\lambda_1} + K^L \circ K^{t,\lambda_2} \ , \tag{2}$$

with K^l the index kernel of level l with output-scale given by σ_l^2, $\forall l \in [\![1, L]\!]$. For simplicity, we also assumed that the lengthscales of the correlation matrices are kept fixed for all levels between 1 and $L-1$ (and set to λ_1), while the lengthscale associated to the replicate level L is allowed to be different (and set to λ_2).

With these notations, the likelihood can be re-intrepreted as follows:

$$\begin{aligned} Y|T, \theta &\sim \mathcal{N}(\mathbf{0}, \left(\sum_{l=1}^{L-1} K^l \right) \circ K^{t,\lambda_1} + \left(K^L \circ K^{t,\lambda_2} + \beta^2 I_N \right)) \\ &\sim \mathcal{N}(\mathbf{0}, \Sigma_m(T,T) + \Sigma_n(T,T)) \ . \end{aligned} \tag{3}$$

Σ_m (with m standing for *model*) describes the correlation captured by modelling the data (typically using the protein and conditions levels in a three level HGP model). Σ_n (with n standing for *noise*) describes the correlation capturing the noise in the data. This noise itself is composed of a correlated part ($K^L \circ K^{t,\lambda_2}$), which captures biological differences between replicates, and an uncorrelated noise ($\beta_p^2 I_N$), modeling measurement errors.

We now consider the problem of defining parameters for a synthetic hierarchical GP model with L_s levels, C_s conditions and R_s replicates in total (with $R_{s,c} = \frac{R_s}{C_s}$ a constant number of replicates per conditions), based on the parameters estimated for this real protein p. The idea is to change the index kernels K^l, via the definition of new values for the output-scales σ_l^2, $\forall l \in [\![1, L]\!]$, while keeping reasonable values for these output-scales. Reasonable values are values which would be biologically plausible. To this aim, we take the following approach: considering one real replicate r, one can summarise the total amount of explained variance for this replicate by:

$$\sum_{l=1}^{L-1} \sigma_l^2 + \sigma_{L,r}^2 + \beta^2 \ . \tag{4}$$

Keeping λ_1, λ_2 and β unchanged for simplicity, we propose to generate synthetic values of $\{\sigma_{l,s}^2\}_{l=1}^{L-1}$ and $\{\sigma_{L_s,r}^2\}_{r=1}^{R_s}$, such that, overall, the amount of explained variance by the model part and the correlated-noise part is maintained:

$$\begin{aligned} \sum_{l=1}^{L-1} \sigma_l^2 &\equiv \sum_{l=1}^{L_s-1} \sigma_{l,s}^2 \\ \sum_{r=1}^{R} \sigma_{L,r}^2 &\equiv \sum_{r=1}^{R_s} \sigma_{L_s,r}^2 \end{aligned} \tag{5}$$

Considering the type II MLE parameters θ_p^{MLE-II} estimated for this real protein p, we can define two quantities: the average amount of explained variance by one level of the

model part $\overline{\sigma_m^2}$, and the average amount of explained variance by one replicate of the correlated-noise part of the model $\overline{\sigma_n^2}$:

$$\begin{aligned}\overline{\sigma_m^2} &= \frac{1}{L-1}\sum_{l=1}^{L-1}\sigma_l^2 \\ \overline{\sigma_n^2} &= \frac{1}{R}\sum_{r=1}^{R}\sigma_{L,r}^2\end{aligned} \tag{6}$$

To be noted, the definition of $\overline{\sigma_n^2}$ allows the output-scale of the lowest level (replicate level) to have a different value for each replicate. This is especially useful to better model replicate to replicate variability and has been typically used in our manuscript.

Using these quantities, we consequently define for the synthetic model the total amount of explained variance by the model part $\sigma^2_{m_s,total}$ and the total amount of explained variance by the correlated-noise part of the model $\sigma^2_{n_s,total}$:

$$\begin{aligned}\sigma^2_{m_s,total} &= \overline{\sigma_m^2}\cdot(L_s-1) \\ \sigma^2_{n_s,total} &= \overline{\sigma_n^2}\cdot R_s\end{aligned} \tag{7}$$

Finally, we randomly split these total amount of explained variances between the different levels (for $\sigma^2_{m_s,total}$) and replicates (for $\sigma^2_{n_s,total}$):

$$\begin{aligned}\sigma^2_{m_s,total} &= \sum_{l=1}^{L_s-1}\sigma^2_{l,s} \\ \sigma^2_{n_s,total} &= \sum_{r=1}^{R_s}\sigma^2_{L_s,r}\end{aligned} \tag{8}$$

The last step consist in defining that $\sigma^2_{2,s} = \max_{l\in[\![1,L_s-1]\!]}\sigma^2_{l,s}$. Indeed, we observed that reproducible differences between conditions are obtained if the output-scale corresponding to the conditions (i.e. level two) is larger than the other output-scales for the model part. Considering the interpretation of the output-scales, this choice is natural, as we wish to generate under the full model observations for which the largest amount of variations is explained by the condition. To be noted, this choice of output-scales also ensures cleaner observations generated under the joint model.

Variability of the synthetic datasets. As requested by the Guest Editor, we tested GPMelt for typical three-level HGP models, but also for deeper hierarchies, like four-level HGP models. Additionally, we investigated the effect of the proportion of true differential melting curves in the synthetic dataset, varying this number from 1% to 30%. Finally, we propose to examine the impact of noise on GPMelt results. Especially, we propose to increase the amount of correlated noise in our dataset using a factor of 1 to 1000. More precisely, this consists in multiplying the total amount of explained variance by the correlated-noise part of the model $\sigma^2_{n_s,total}$ (eq (7)) by this factor before splitting the result between replicates. This models the existence of proteins presenting some strong outlier replicates. Finally, we also explored the effect of fixing (or not) the value of the output-scale of the replicate level, i.e having the same value $\sigma^2_{L_s}$ for all replicate, or allowing for a different value $\sigma^2_{L_s,r}$ for each replicate r.

In summary, we tested the following configurations (120 configurations in total, with $N = 1000$ IDs for each configuration and $S = 10$ samples per ID for each scenario).

1. Scenarios : HGP models used to generate and subsequently to fit the synthetic data. Short names in bold are the ones used in Figures presenting the results (S3 to S12 Figs, p.32 to 41 of the supplement text).

- **3Lev - Fixed lev 3 - 2 Cds - 2 reps** : three-level HGP model with two conditions, two replicates for each condition and the replicates' output-scale kept fixed.
- **3Lev - Free lev 3 - 2 Cds - 2 reps** : three-level HGP model with two conditions, two replicates for each condition and the replicates' output-scale allowed to vary between replicates.
- **3Lev - Fixed lev 3 - 3 Cds - 2 reps** : three-level HGP model with three conditions, two replicates for each condition and the replicates' output-scale kept fixed.
- **3Lev - Free lev 3 - 3 Cds - 2 reps** : three-level HGP model with three conditions, two replicates for each condition and the replicates' output-scale allowed to vary between replicates.
- **4Lev - Fixed lev 4 - 2 Cds - 2 reps** : four-level HGP model with two conditions, two sub-groups, two replicates for each sub-group and the replicates' output-scale kept fixed.
- **4Lev - Free lev 4 - 2 Cds - 2 reps** : four-level HGP model with two conditions, two sub-groups, two replicates for each sub-group and the replicates' output-scale allowed to vary between replicates.

2. Noise factor: factor used to multiply $\sigma^2_{n_s,total}$ (eq (7)) before being split between replicates:
   - $\{1, 10, 100, 1000\}$
3. Fraction of true differential IDs:
   - $\{0.01, 0.05, 0.1, 0.2, 0.3\}$

Results:

- We start by depicting examples of 10 samples (five true differential and five true negative IDs) for each scenario with different noise levels in S3 to S6 Figs (p.32 to 35 of the supplement text).
- We then discuss the resulting ROC curves (S7 Fig, p.36 of the supplement text). The columns depict the different scenarios, the rows the different noise factors. The colors of the curves indicate the fraction of true differential IDs. From this figure, it can be seen that, up to a factor of 100, GPMelt allows to recover almost perfectly all true differential IDs. The performance of GPMelt only starts to drop for a noise factor of 1000, while remaining pretty good in most scenarios. It can be noted that in this case and for the scenarios with three conditions, a very low amount of differential IDs (e.g. a fraction of 0.01, i.e only 10 true differential IDs out of 1000) can strongly impact the performance of GPMelt. It is also important to account for the fact that using a noise factor of 1000 leads to simulate a real dataset with an extremely high level of noise, as shown in S6 Fig (p. 35 of the supplement text), which depicts some samples obtained at this noise level for the different scenarios.
- Subsequently, we investigate the FDR control by plotting the expected vs observed FDR in S8 Fig (p. 37 of the supplement text). Here also, the columns correspond to the different HGP models' specifications, the rows to the different noise factors and the colors to the fraction of true differential IDs. The black line indicates the $y = x$ line, which represents the ideal case. We see from this figure

that GPMelt controls the FDR accurately in most of the cases and up to a noise factor of 100. For a noise factor of 1000, the FDR control can not be guaranteed anymore. It can also be noted that for smaller fractions of true differential IDs (typically 0.1), the observed FDR is generally larger than the expected FDR.

- Finally, we present the Quantile-Quantile plots (QQplots) showing the theoretical quantiles of a uniform $(0, 1)$ distribution vs the empirical quantiles computed from the p-values obtained using GPMelt. S9 to S12 Fig (p.38 to 41 of the supplement text) show the QQplots obtained for the different scenarios (columns) and different fractions of true differential IDs (rows). Each plot corresponds to a different noise factor. Points following the black line $y = x$ correspond to p-values uniformly distributed, while points diverging from this line are p-values diverging from the uniform assumption. Well-calibrated p-values are obtained when the negative IDs are associated to p-values uniformly distributed, and hence lying along the $y = x$ line, and the differentials IDs have p-values diverging from the $y = x$ line. As the fraction of differential IDs increases, we expect more points to diverge from the $y = x$ line. To be noted, the empirical computation of the p-values has a precision limited by S, the amount of samples in the null distribution approximation, and can't be smaller than

$$\min p = \frac{1}{1+S} \ . \tag{9}$$

Hence, as the number of differential IDs increases in our synthetic datasets, we expect more p-values to tend towards or equal this minimum. Globally, these QQplots show that the p-values are well calibrated for a noise factor of 1 and 10, and remain mostly acceptable for larger noise factors.

Conclusion: We showed that GPMelt provides well-calibrated p-values for all scenarios at normal noise level. We also showed that p-values calibration is rather robust to an increase of noise, testing scenarios 10 to 1000 times more noisy than a clean dataset. We also demonstrated that the FDR is well controlled for datsets up to 100 times more noisy than real clean dataset, and ROC curves illustrate the accuracy of GPMelt to detect true differential IDs in the 120 different configurations we tested. We added the description of the synthetic dataset generation and of the results as S3 Supporting Information, and the results figures S3 to S12 in the supplement text. Additionally, the following modifications of the **Results** section in the main text have been made:

- l.378-377 (p.13 of the main text) have been modified as follows: "We start by showing the validity of GPMelt framework using a simulation study. Subsequently, to benchmark the GPMelt framework with a three-level HGP model on real data, we reanalysed [...]"
- The following paragraph has been added at l.393-410 (p.13-14 of the main text): "**Simulation study**: To validate the newly introduced statistic Λ, we proceed to a simulation study, in which we generated synthetic data according to GPMelt full and joint models, for hierarchical models with three or four levels, and with two or three conditions. Additionally, we investigated the effect of the proportion of true differential melting curves in the synthetic dataset, varying this number from 1% to 30%. Finally, we examined the impact of noisy data on GPMelt results, and increased the amount of correlated noise per replicate from 1 to 1000 times more than observed in clean real data. Example of samples forming the different synthetic datasets are depicted in S3 to S6 Figs.

We start by demonstrating the accuracy of GPMelt to detect true differential melting curves using ROC curves (S7 Fig). Subsequently, we show that the FDR is well controlled for datsets up to 100 times more noisy than real clean data (S8 Fig). Finally, we illustrate that the use of Λ and the sampling approach to approximate its null distribution provides well-calibrated p-values at normal noise level, calibration which remains fairly robust to an increase of noise (see S9 to S12 Figs). S3 Supporting information provides additional descriptions regarding the synthetic dataset generation and the obtained results."

Reviewer 1:

In this paper, the authors used a hierarchical Gaussian process model to study variations in the melting profiles of proteins resulting from different treatments and multiple replications. The proposed nonparametric model, adapted from Hensman et al. 2013, does not impose any shape constraints on the melting curves. This modelling choice improves the flexibility of the model, enabling it to capture non-conventional patterns. The research is presented clearly, and the results are promising. However, I have a few questions that should be addressed prior to publication.

Comment 1

1. The proposed method does not impose positivity constraints on the hierarchical prior of the melting curves. As a result, not all elements in the parameter space have biologically sensible interpretations (e.g., the 95% credible band of a melting curve may include negative intensities). How should users interpret the results in such cases, and is this a concern in real-world applications?
We agree with the reviewer that the proposed method does not impose positivity constraints on the hierarchical prior of the melting curves. As a results, the mean or 95% credible band of the predictive distribution can include negative values, which would consequently not be biologically interpretable.
A possible solution would be to impose a non-linear link function on the bottom layer of our hierarchy. More precisely, we could update the three-level HGP model (and similarly the four-level HGP model) as follows:

$$\begin{array}{l} \forall\ i \in [\![1, N]\!] \\ \forall\ r \in [\![1, R]\!] \\ \forall\ c \in [\![1, C]\!] \end{array} \begin{cases} h \sim GP(0, k_h(t, \cdot|\lambda_1)) & \textit{protein} \\ g_c \sim GP(h, k_g(t, \cdot|\lambda_1)) & \textit{conditions} \\ f_{cr} \sim GP(g_c, k_{f_{cr}}(t, \cdot|\lambda_2)) & \textit{replicates} \\ y_{cri} = invlink(f_{cr}(t_i)) + \epsilon_i & \textit{observations} \\ \epsilon_i \overset{iid}{\sim} \mathcal{N}(0, \beta^2) & \end{cases} \tag{10}$$

with $invlink$ the inverse of a function such that $invlink\colon \mathcal{R} \to [0, +\infty[$. Examples of such function could be:

- the logistic function: $invlink(x) = \frac{L}{1+e^{-k(x-x_0)}}$, with $L, k, and x_0$ some parameters,
- the CDF (cumulative distribution function) of the standard normal distribution: $invlink(x) = \frac{1}{\sqrt{2\pi}} \int_{-\infty}^{x} \exp(-\frac{u^2}{2}) du.$

While ensuring positivity constraints on the melting curves, this approach would have multiple drawbacks:

- The interpretability of the last level of the hierarchical model would be lost, due to the use of a non-linear link function. As described in the manuscript, the output-scales of the last level of the hierarchy measure replicate-wise deviations from the condition-wise melting curves, and hence provide a convenient proxy to outliers detection.
- Due to the lost of linearity, the likelihood of the model wouldn't be Gaussian anymore (see Eq (9) of the manuscript). GP regression with non-Gaussian likelihood involves computation of intractable integrals, e.g. when computing the marginal likelihood. Hence, GP regression with non-Gaussian likelihood requires non-exact methods like Monte Carlo sampling based or variational approximation of integrals (we refer to [4] for a general and complete introduction to the problem). These estimations methods are either computationally more expensive that the exact type II MLE algorithm used in the present work (in the case of MCMC) or provide reduced modelling accuracy.
- Moreover, the proposed framework is based on a null distribution estimation build with samples from the joint model. Here also, a non-Gaussian likelihood would greatly increase the cost of the null distribution estimation.

In theory, we thus agree that imposing positivity constraints on the proposed GP regression would improves the rigor of the model and the biological interpretation of the melting curves. In practice, the hierarchical model itself, by constraining the fits of all replicates based on other replicates already favors smooth estimates of the melting curves, rather than rapidly oscillating melting curves (with high probability of crossing the x-axis). Moreover, if the melting curves estimate is not smooth enough, constraints on the lengthscale can be enforced. This has typically been done in this work, where the lengthscale was forced to be larger than (1) the minimum or (2) the maximum distance between successive temperatures in a replicate.
To conclude, we agree that the suggestion of imposing positivity constraints on the hierarchical prior of the melting curves would improve the rigor of the modelling, but we argue that the induced computational cost, being substantial, made us favor a simpler model. Moreover, the probability of observing negative values for the mean or 95% credible band of the predictive distribution is kept rather low, thanks to the constraints imposed by the hierarchical model and the additional constraints that can be enforced on the kernels lenghtscale.

Acknowledging the accuracy this comment, we added this sentence to the discussion (l.807-812, p.22 of the main text)

- "A possible limitation of this model is the lack of positivity constraints on the melting curves. Adding these constraints via a link function would have substantially increased the computational cost of the framework, while reducing the model interpretability. In practice, the constraints imposed by the hierarchical model and the possible additional constraints on the lengthscale typically minimise the probability of predicted negative values."

Comment 2

2. Likelihood ratio test and B-H correction are used in testing the effect of different conditions. Please explain better the motivation of the use of LR test over other selection criteria such as e.g. BIC.
We thank the reviewer for this very interesting comment, and explain in the following the choices made in GPMelt framework.
GPMelt corresponds to an hypothesis testing framework (tending towards frequentist approach) rather than a model selection framework (Bayesian approach). In our

framework, the null hypothesis states that the treatment has no effect on the protein (which translates in not affecting the protein's melting behaviour). We aim to find the proteins in our sample for which the data present enough evidence to reject this hypothesis. For these proteins, the melting behaviour in the treatment condition will be considered significantly different from the protein's melting behaviour in the control condition.
Considering a protein p with observations denoted by (T_p, Y_p), we proceed as follows (a visualisation of these steps are proposed in the new version of Fig 1, panel B from the main text, and the new S1 Fig of the supplement text):

- After observing the data (T_p, Y_p) (measurement of TPP-TR experiments), we model the data using a hierarchical Gaussian process (HGP) model that takes into account the different conditions. This model is called full model, and denoted by $\mathcal{M}_1$, as it fully describe each condition as a separate task in the HGP model. This model will help us to test the *alternative* hypothesis, i.e. that the protein's melting behaviour between the two conditions are actually different.

- We use this model $\mathcal{M}_1$ to estimate the parameters θ_p^{MLE-II} via type II MLE (which optimises the *marginal* likelihood).

- Consequently, we define a joint model $\mathcal{M}_0$, which is identical to the full model $\mathcal{M}_1$ up to a change in the covariance matrix structure. The structure of the joint covariance matrix models the assumption that the control and treatment conditions can be modelled together as a shared task, i.e. we do not assume differences in protein's melting behaviour between these two conditions.

- Parameters of this joint model $\mathcal{M}_0$ would not be identifiable, hence we propose to fix the parameters of this joint model to the parameters θ_p^{MLE-II} estimated for the full model $\mathcal{M}_1$.

- Using the set of parameters θ_p^{MLE-II}, we compute the marginal likelihood of the observations under these two models $\mathcal{M}_0$ and $\mathcal{M}_1$.

- We next define a statistic, that we call Λ_p as follows :

$$\Lambda_p = -2 \cdot \log \left(\frac{p(Y_p | T_p, \theta_p^{MLE-II}, \mathcal{M}_0)}{p(Y_p | T_p, \theta_p^{MLE-II}, \mathcal{M}_1)} \right) . \tag{11}$$

  where (T_p, Y_p) are the observations for protein p, $\mathcal{M}_0$ stands for joint model and $\mathcal{M}_1$ for full model. It can be noticed that this statistic is *similar but different* from a Likelihood Ratio test statistic, because the models $\mathcal{M}_0$ and $\mathcal{M}_1$ share the same set of hyper-parameters θ_p^{MLE-II}. The model $\mathcal{M}_0$ is *never* fitted to the data. A Likelihood Ratio test statistic requires both models $\mathcal{M}_0$ and $\mathcal{M}_1$ to be fitted via MLE.

- We then proceed to the approximation of the null distribution of our statistic Λ_p, because the analytical form of the null distribution of this statistic is unknown. The null distribution approximation is constructed by a sampling approach, using the generative joint model $\mathcal{M}_0$ with parameters fixed to θ_p^{MLE-II}.

- Once an appropriate number S of null samples have been drawn from the generative joint model, the full model $\mathcal{M}_1$ is fitted to each of these samples in order to compute the value of Λ_s^0 for each sample s. Combining the obtained values of $\{\Lambda_s^0\}_{s \in S}$ forms the null distribution approximation for our statistic Λ_p.

- Using this null distribution approximation, we can compute a p-value p_r associated to the statistic Λ_p.

The previous procedure is repeated for all proteins p of the dataset. In the manuscript, we propose different versions of the null distribution approximation of the statistic Λ, and the one described here is one of them. Hence, we are given a set of p-values, one for each protein p. We further proceed to a multiple testing correction using the Benjamini-Hochberg (BH) procedure [5]. This procedure has mainly been chosen to simplify the comparison with the previously published results of the two methods we compare GPMelt with (namely NPARC [3] and the Bayesian semi-parametric model [1]). Other FDR control approaches, like the q-value [6], could be applied if preferred.
Our choice of statistic Λ was guided by the following main reasons (see S2 Supporting Information for in-depth discussion):

- This statistic is interpretable: it is the log ratio of the marginal likelihood of the data under the two models we aim to compare. A large value of this statistic carries evidences against the null hypothesis, as this means that the marginal likelihood of the data under the full model is considerably larger than under joint model.

- The *log marginal likelihood* of the data under model $\mathcal{M}_i$, denoted $\log p(Y_p|T_p, \theta_p^{MLE-II}, \mathcal{M}_i)$ is a key quantity for the estimation of the model parameter via type II MLE. As such, it is readily available in most libraries performing GP regression.

- In the manuscript, we link this statistic to a previously published work [7] where a similar statistic was shown to be an appropriate similarity measure for time series modeled by GPs.

The use of the *BIC* criterion in the current GPMelt framework would not be appropriate, as it requires the maximised value of the likelihood of the data for each model. As described above, the type II MLE is only used to estimate the parameters of the full model. Parameters of the joint model are *not* estimated.

We thank the reviewer for this question, and propose the following modifications to improve the clarity of the manuscript:

- Fig 1 (p.4 of the main text) and S1 Fig (p.30 of the supplement text) have been updated to improve the clarity about GPMelt procedure and algorithmic steps.

- We modified l.74 to 77 (p.3 of the main text) by "In this work, we propose to model melting curves via a hierarchical model based on Gaussian Processes (GPs), and to assess significance of differentially melting curves using a statistic, denoted hereafter by Λ, whose distribution under the null hypothesis has to be approximated. "

- We updated the section about **Hypothesis testing framework** (p.10 to 11 of the main text) to emphasize that GPMelt statistic Λ is not a Likelihood Ratio statistic, and do not share the same properties.

- We added a simulation study to this work to demonstrate the validity of the statistic Λ, and added this simulation study as a paragraph in the Results section (l.393-410, p.13 to 14 of the main text), and as S3 Supporting Information with new figures S3 to S12 Figs).

- We updated the S2 Supporting Information to further describe the statistic Λ and its properties, and to emphasize the dissimilarities with a standard Likelihood Ratio test statistic.
- We also corrected the S1 Appendix: "Bayesian model inference and selection", where some notation about the Bayes factor and the marginal likelihood were incorrect.

Comment 3

3. Does the posterior inference of mean functions associated with different layers (e.g. $h, g, f, \dots$) suffer from identifiability issues as the depth of hierarchies increases?

We thank the reviewer for this question about identifiability issues with increasing depth of the hierarchy. First of all, we would like to clarify that the posterior inference of the mean functions associated with different layers (e.g. $h, g, f, \dots$) are not independent from each other. The full model, which includes all layers of the hierarchy, is fitted using type II MLE. The estimated parameters are then used to proceed to the posterior inference of each level. More precisely, if we consider the three-level hierarchical model (Eq (8) of the main) restated hereafter:

$$\begin{array}{l} \forall\ i \in [\![1, N]\!] \\ \forall\ r \in [\![1, R]\!] \\ \forall\ c \in [\![1, C]\!] \end{array} \left\{ \begin{array}{ll} h \sim GP(0, k_h(t, \cdot|\lambda_1)) & \textit{protein} \\ g_c \sim GP(h, k_g(t, \cdot|\lambda_1)) & \textit{conditions} \\ f_{cr} \sim GP(g_c, k_{f_{cr}}(t, \cdot|\lambda_2)) & \textit{replicates} \\ y_{cri} = f_{cr}(t_i) + \epsilon_{cri} & \textit{observations} \\ \epsilon_{cri} \overset{iid}{\sim} \mathcal{N}(0, \beta^2) & \end{array} \right. \tag{12}$$

The posterior inference is done as follows:

- for the bottom level, we use the HGP model with all levels: $y_{cri} = f_{cr}(t_i) + \epsilon_{cri}$,
- for the middle level, we remove the modeling provided by the bottom level of the HGP model, and use $y_{cri} = g_c(t_i) + \epsilon_{cri}$ for the predictions,
- for the top level, we only consider the function h to predict the observations and use $y_{cri} = h(t_i) + \epsilon_{cri}$.

This can be interpreted as removing, for each level, all the additional information on the covariance structure provided by the lower levels.

Regarding identifiability issues: As the depth of the hierarchy increases, the model becomes more complex, and multiple local optima become more likely. While this is typically not a problem for the protein-level datasets, we encountered this limitation with the peptide-level dataset (phosphoTPP dataset [8]). To solve this issue, we propose to run the optimization multiple times for each protein and select the best optimum, whose parameters are then used for the null distribution estimation and statistic evaluation. This is mentioned in S3 Table (p.44 of the supplement text), describing the model specifications of the benchmarking dataset: "For very complex models, multiple optima could exist. Hence, the fitting of the observations for each protein has been estimated three independent times, and the parameters leading to the best fit (lowest final loss value from the type II MLE estimation) are selected for the null distribution sampling and the rest of the analysis."

Comment 4

4. How does the proposed method scale with the size of the dataset? It would be helpful if the authors could provide relevant metrics such as wall clock time or number of function calls.

We thank the reviewer for this suggestion, and added the supplementary figure S23 Fig. We refer to this figure in the section **Code availibility** (p.833-834, p.23 of the main text):

- "An overview of the resource requirement of the current implementation is illustrated in S23 Fig for the ATP 2019 and phosphoTPP datasets."

Reviewer 2

Le Sueur et al. present an important update to the analytical pipelines for TPP. TPP is already an invaluable helpful technology for an unbiased analysis of drug targets. Hence an extension to the analytical toolkit that expands the number of proteins that can be statically evaluated within an experiment would of value to the community, especially if it can scale to multiple conditions as experiments with multiple concentrations, cell lines and temperature points become more commonplace. The outlier control within the model is also a valuable addition, as having one malfunctional biological replicate could considerably compromise the results.

Comment 1

It is not clear how to obtain, download or install GPMelt and the readme file doesn't not provide any guidance or assistance. Considering the main point of the manuscript is a tool for the community, this seems a major issue.

We thank the reviewer for this comment, and have updated the content of the gitlab repository. The code to reproduce the analyses and figures are now available, along with a Nextflow pipeline based on our GPMelt implementation in python.

- The section **Code availability** (p.827-834, p.23 of the main text) has been changed to the following: "The method implementation along with the code to reproduce the analyses and figures are available on the following gitlab repository: `https://git.embl.de/grp-savitski/gpmelt.git`. The method implementation is provided in Python, accompanied by a Nextflow pipeline. This setup ensures simple installation, supports parallel computation, and offers a portable solution for deployment on both local computers and high-performance computing (HPC) clusters."

- We added a tutorial on the gitlab repository to explain how to install and run the Nextflow pipeline.

Comment 2

2. The solution is presented as based on TMT where this technology is consistently losing ground to label free DIA approaches. Does GPMelt work with label free DIA and if not this would be an important method to maximise its value.

We appreciate the reviewer's insightful comment, and justify hereafter why GPMelt would also be suited for label free DIA, along with any type of continuous data measured across possible multiple conditions. While GPMelt has been originally developed to work with TPP datasets generated using TMT, the model itself only requires continuous data: any quantitative values measured at different temperatures or

time points (or any type of continuous variable), coming from two to multiple conditions and generated by any type of quantitative methodology could be fitted and analysed using GPMelt framework.

To make it clearer to the reader, we added the following sentence in the discussion (l.813-816, p.22 of the main text):

- "Importantly, while originally developed to improve TPP-TR dataset analyses generated using TMT, the versatile and general framework of GPMelt extends beyond TPP-TR analysis and could be applied to any continuous datasets by quantitative methods (e.g DIA) presenting replicates and conditions."

Comment 3

3. Related to the approximate ROC curve. How likely is the treatment to inhibit all potential targets at the concentration? This is very likely to be uneven and impossible to predict, therefore this measure seems quite optimistic more than a realistic analysis. (line 385 onwards)
We thank the reviewer for this comment, and justify hereafter the use of approximate ROC curves to compare different methods. These approximate ROC curves are not meant to capture the absolute efficiency of our method, but instead provides a useful framework to compare the *relative performance* of different methods. Similar approximate ROC curve analyses were performed in the original NPARC [3] (see Fig 5) and Bayesian semi-parametric model [1] (see Fig 2 B) papers.
Moreover, we believe that this analysis is typically pessimistic instead of optimistic, because the true negatives are not well defined [1]. While it is true that not all targets are expected to be inhibited (meaning that the sensitivity will not reach one), we also count as "false positive" any other not expected/not known target proteins that might truly be affected by the treatment (hence reducing the apparent specificity of the method). Typically, methods which capture more hits are likely to be more strongly penalized on their specificity. Hence, to be fair to the Bayesian semi-parametric model, which presents a very low specificity compared to GPMelt on the ROC curves (Fig 4A of the main text), we investigated the reason for this lack of specificity. We illustrate with Fig 6AB (main text) that the Bayesian semi-parametric model is actually less specific than GPMelt due to its sensitivity to outliers. To conclude, with the approximate ROC curve analysis, we showed that GPMelt is as sensitive and almost as specific than NPARC, while being more strongly penalized for the specificity, as GPMelt typically captures more hits than NPARC at a given α- threshold on the adjusted BH p-value.

The following sections of the manuscript justify our approach:

- (l.418-427, p.14-15 of the main text): "To proceed to the methods comparison, an *approximate* receiver operator characteristic (ROC) curves is built for each method. The term *approximation* refers to the exact ground truth being unknown, but approximated by the set of proteins expected to be targeted by the treatment. However, proteins in this set are unlikely to all show a significant change in melting behaviour. Furthermore, some proteins which don't belong to this set (either due to incorrect annotation or because they are unexpected (off-)targets of the investigated compounds) might as well present important variations in their melting curves. Taking this into consideration, the set of expected target proteins can be defined using the Gene Ontology (GO) Consortium annotations curated in Uniprot [9]."

- (l.438-442, p.15 of the main text): "In addition, while the Bayesian semi-parametric model also uses GPs to relax the sigmoidal assumption, the ROC

curve obtained for this method on the Staurosporine 2014 dataset highlights the lack of specificity of this model compared to GPMelt (similarly illustrated in Fig S15A). This point is further investigated in a subsequent section about outliers detection. ”

- (l.634-639, p.19 of the main text): “However, a cautious examination of their hits led us to conclude that this method might also suffer from a significant sensitivity to outliers. To show this, we considered the Staurosporine 2014 dataset and selected the proteins for which the BH adjusted p-values from both NPARC and GPMelt were greater than 0.8, while being captured as significant hits by the Bayesian semi-parametric model at an α-threshold of 0.01.”

Comment 4

4.Authors should clearly acknowledge the limitation of determining the specific phosphorylation site when using bottom-up proteomics for their site specific GPMelt analysis.

We thank the reviewer for this comment. Indeed, when using bottom-up proteomics, one can only determine the phosphorylation status for the observed peptides, but not for the full protein. Likewise it is not possible to know if phosphorylation sites on two distinct peptides occur simultaneously or independently of each other. This facts motivates our ”phospho-site analysis” (l.560-628, p.17-19 of the main text), in which we suggest to only compare peptides for which the phosphorylation status is fully comparable. However, it is true that this analysis does not solve the issue that the phosphorylation state of the rest of the protein which the peptides are coming from can not be resolved. Hence, the observed melting behaviour of a peptide might be due to PTMs located outside of the peptide itself, and not to the PTMs observed on the peptide under study.

We added the following sentence l.625-628 (p.19 of the main text) to acknowledge this limitation:

- “Further follow-up experiments would be required to validate these hypotheses, as the present analysis cannot exclude the impact of unseen phosphorylation sites outside of the sub-sequence of interest on the melting behaviour of the peptides.”

Comment 5

5.Mean intensities are subject to bigger effects, especially in low replicate counts as expected in TPP experiments, with the median being far more robust why was this not used?

We thank the reviewer for this insightful comment, and justify our choice of mean scaling over median or geometric mean scaling. Using the newly added Supplementary Figures S21 and S22 Figs, we show that the median scaling is not appropriate in the case of melting curves, as the median of a replicate is too sensitive to the shape of the curve, and particularly to the speed of the melting (represented by the slope of the curve when the shape is sigmoidal). Moreover, we show that the geometric mean, usually more appropriate than the arithmetic mean for times series data, is too sensitive to very low values, that are typically observed in the melting curves at low temperatures. Comparatively, the mean scaling is more robust to the curve shape and less affected by very small intensity values, hence making this scaling a preferred alternative to the FC scaling.

We have added S21 and S22 Figs (p.55-56, supplement text) and updated the S5 Supporting Information as follows:

- We updated the introduction of the section (p.25-26, supplement text):
  "In the main text, we introduced a new scaling of the observations to replace the broadly used Fold Change scaling. Hereafter, we further detail the advantages and drawbacks of the Fold Change scaling, and illustrate how the mean scaling addresses the main limitations of Fold Change scaling. We then show that the mean scaling is more appropriate than the median or geometric mean scaling in the case of melting curves."

- We split the section in three subsections :

  1. "The mean scaling as a solution to the limitations of the FC" (p.26-27, supplement text)
  2. "The mean scaling is more appropriate than the median or geometric mean scalings." (p.27-28, supplement text)
  3. "The mean, median and geometric mean scaling of FC data are equivalent to the mean, median and geometric mean scaling of the raw data." (p.28-29, supplement text)

- The following sentence has been added at the end of the first subsection:
  "In the next paragraph, we further justify the choice of the mean scaling compared to the median or geometric mean scaling." (p.27, supplement text)

- The newly added second subsection (p.27-28, supplement text) reads as:
  "In this work, we propose to scale the observations in a replicate by the mean intensity of this replicate. However, the mean is not the only summary statistic that could have been considered. Typically, the median intensity could be more robust to very extreme observations. Also, the geometric mean is often used as replacement for the arithmetic mean in time series analysis. In the following, we investigate the use of the median and geometric mean as scaling factors, and illustrate why the mean scaling is better suited for our data type than the median and geometric mean scaling. To this aim, we use the ATP 2019 dataset [2]. The pre-processed data provided by this paper are already scaled using the Fold Change. We show hereafter (see following subsection) that the mean, median and geometric mean scaling of FC data are equivalent to the mean, median and geometric mean scaling of the raw data. For this reason, we can use FC data equivalently to raw data to demonstrate the advantages of the mean scaling over the median and geometric mean scaling.

  The median is usually used as a more robust estimator of centrality than the mean, which is more sensitive to extreme values. This is typically true for skewed data distribution, which is not the case of TPP datasets. In S21 Fig panel A, we show that the distribution of the FC values are U-shaped. Hence, because the median is approximately the *middle* value of a replicate, this value will be extremely sensitive to the shape of the melting curve. As illustrated in S21 Fig panel B, if one replicate decreases faster than the other, the median value will be much lower than for the other replicate. Hence, the median of each replicate of a condition might end up being very different, leading to scaled replicates looking quite distinct (see S21 Fig panel C, third facet). More generally, we show in S22 Fig panel A that the spread of computed median values (over all replicates and conditions) is much larger than for the mean values. The mean value is less sensitive to differences in global shape of the melting curves, as it takes into account all values along the curves.

  Concerning the geometric mean, the impact of the lowest values of the curves, which might be very small, leads the geometric mean to be potentially very small

compared to the other summary statistic (see S22 Fig panel A). Diving the observations by a small number make the scaled replicates look like *exploding* (see S21 Fig panel C, last facet). In S21 Fig panel B, we show that the absolute difference in scaling factors computed for replicates of a same condition tends to be significantly smaller when using the mean, compared to the median or the geometric mean, as scaling factors (one-sided Wilcoxon rank sum test, p-values $< 2.2e-16$).

Taken together, we have shown that the median scaling is not appropriate in the case of melting curves, as the median of a replicate is too sensitive to the shape of the curve, and particularly to the speed of the melting (corresponding to the slope of the curve when the curve is sigmoidal). Moreover, we have shown that the geometric mean, usually more appropriate than the arithmetic mean for times series data, is too sensitive to very low values, that are typically observed in the melting curves at low temperatures. Comparatively, the mean scaling is more robust to the curve shape and less affected by very small intensity values, hence making this scaling a preferred alternative to the FC scaling."

- The newly added third subsection (p.28-29, supplement text) reads as:
  "In this subsection, we show that the mean, median and geometric mean scaling of FC data are equivalent to the mean, median and geometric mean scaling of the raw data, justifying the use of the ATP2019 dataset in S21 and S22 Figs.

  According to our notations, the scaled intensities are given by:

$$\begin{aligned}
&\forall \quad scaling \in \{FC, mean, median, geomMean\},\\
&(y_i^{cr})^{scaling} = \frac{\gamma_i^{cr}}{\rho_{cr}^{scaling}}\\
&\text{with} \quad \rho_{cr}^{FC} = \gamma_1^{cr}\\
&\rho_{cr}^{mean} = \frac{1}{N_{cr}}\sum_{i=1}^{N_{cr}}\gamma_i^{cr}\\
&\rho_{cr}^{median} = \begin{cases} \gamma^{cr}_{\frac{N_{cr}+1}{2}} & \text{if } N_{cr} \text{ is odd}\\ \dfrac{\gamma^{cr}_{\frac{N_{cr}}{2}} + \gamma^{cr}_{\frac{N_{cr}}{2}+1}}{2} & \text{if } N_{cr} \text{ is even}\end{cases}\\
&\rho_{cr}^{geomMean} = \exp\left(\frac{1}{N_{cr}}\sum_{i=1}^{N_{cr}}\log\gamma_i^{cr}\right)
\end{aligned} \tag{13}$$

  We then denote by $(\rho_{cr}^{scaling})^{FC}$ the scaling computed from the FC instead of the raw intensities, with $scaling \in \{mean, median, geomMean\}$. We have:

$$\begin{aligned}
(\rho_{cr}^{mean})^{FC} &= \frac{1}{N_{cr}}\sum_{i=1}^{N_{cr}}(y_i^{cr})^{FC}\\
&= \frac{1}{N_{cr}}\sum_{i=1}^{N_{cr}}\frac{\gamma_i^{cr}}{\rho_{cr}^{FC}}\\
&= \frac{1}{N_{cr}\cdot\gamma_1^{cr}}\sum_{i=1}^{N_{cr}}\gamma_i^{cr}\\
&= \frac{\rho_{cr}^{mean}}{\gamma_1^{cr}}\ .
\end{aligned} \tag{14}$$

Similarly, we have:

$$(\rho_{cr}^{median})^{FC} = \begin{cases} (y_{\frac{N_{cr}+1}{2}}^{cr})^{FC} & \text{if } N_{cr} \text{ is odd} \\ \frac{(y_{\frac{N_{cr}}{2}}^{cr})^{FC} + (y_{\frac{N_{cr}}{2}+1}^{cr})^{FC}}{2} & \text{if } N_{cr} \text{ is even} \end{cases} \tag{15}$$

$$= \frac{\rho_{cr}^{median}}{\gamma_1^{cr}} \ ,$$

and

$$\begin{aligned} (\rho_{cr}^{geomMean})^{FC} &= \exp\left(\frac{1}{N_{cr}}\sum_{i=1}^{N_{cr}} \log\left((y_i^{cr})^{FC}\right)\right) \\ &= \exp\left(\frac{1}{N_{cr}}\sum_{i=1}^{N_{cr}} \log \frac{\gamma_i^{cr}}{\rho_{cr}^{FC}}\right) \\ &= \exp\left(\frac{1}{N_{cr}}\sum_{i=1}^{N_{cr}} \left(\log \gamma_i^{cr} - \log \rho_{cr}^{FC}\right)\right) \\ &= \exp\left(\frac{1}{N_{cr}}\sum_{i=1}^{N_{cr}} \log \gamma_i^{cr}\right) \cdot \exp\left(\frac{-1}{N_{cr}}\sum_{i=1}^{N_{cr}} \log \rho_{cr}^{FC}\right) \\ &= \rho_{cr}^{geomMean} \cdot \exp(\log(\frac{1}{\gamma_1^{cr}})) \\ &= \frac{\rho_{cr}^{geomMean}}{\gamma_1^{cr}} \ . \end{aligned} \tag{16}$$

Thus, the scaling of FC data is equivalent to the scaling of the raw data:

$$\begin{aligned} ((y_i^{cr})^{FC})^{mean} &= \frac{(y_i^{cr})^{FC}}{(\rho_{cr}^{mean})^{FC}} = \frac{\gamma_i^{cr}}{\gamma_1^{cr}} \cdot \frac{\gamma_1^{cr}}{\rho_{cr}^{mean}} = (y_i^{cr})^{mean} \\ ((y_i^{cr})^{FC})^{median} &= \frac{(y_i^{cr})^{FC}}{(\rho_{cr}^{median})^{FC}} = \frac{\gamma_i^{cr}}{\gamma_1^{cr}} \cdot \frac{\gamma_1^{cr}}{\rho_{cr}^{median}} = (y_i^{cr})^{median} \\ ((y_i^{cr})^{FC})^{geomMean} &= \frac{(y_i^{cr})^{FC}}{(\rho_{cr}^{geomMean})^{FC}} = \frac{\gamma_i^{cr}}{\gamma_1^{cr}} \cdot \frac{\gamma_1^{cr}}{\rho_{cr}^{geomMean}} = (y_i^{cr})^{geomMean} \end{aligned} \tag{17}$$

”

Comment 6

6.Also by normalising all values by the mean, the differences between control and treatments are artificially being compressed. Only relative measure will be compared when large absolute differences could be masked.

We thank the reviewer for this question, and would like to clarify that the scaling of the raw data is a requirement for all current statistical analysis developed for TPP-TR datasets. For NPARC [3] (and to some extend to the Bayesian semi-parametric model [1]), the definition of the sigmoid used to fit the data (Eq (5) p.6 of the main text) imposes the value at the lowest temperature to be one. Hence the use of FC scaling which ensures this point. For GPMelt, the scaling of the observations is justified by the use of an optimization algorithms based on gradient descent, whose fast convergence requires the input data to all be in a similar range. The following sentences of the S5 Supporting Information (p.26 of the supplement text) explain the same point:

NPARC "Finally, fixing the initial scaled intensity to one simplifies the fitting of the sigmoid function S, with $S_\infty = \lim_{T \to -\infty} S(T) \equiv 1$ being a fixed parameter. "

GPMelt "Theoretically, the model could thus directly work on raw intensities, concurrently fitting melting curves (i.e tasks) and estimating tasks specific scaling factors, with the scaling factor indicating by how much each replicate melting curve (lowest level of the hierarchy) is *vertically* shifted from the common trend (second last level of the hierarchy). However, in practice, raw intensities measured from different MS runs can have several order of magnitudes differences. For this reason, scaling raw intensities beforehand helps to bring replicates observations to a similar range, which is typically recommended when using optimization algorithms based on gradient descent (the current GPMelt implementation with GPyTorch [10] uses the Adam algorithm [11]). In this way, the optimization algorithm is converging faster towards the minima. "

Minor comments

- Font sizes in figure 1 are not too small, especially the equations. We thank the reviewer for this comment and have updated the figure.
- Figure 3 graph legend font sizes are far too small to read. We thank the reviewer for this comment and have updated the figure.
- Figure 4 requires much larger font size on the legends, having too many horizontal panels makes it compressed and hard to read and interpret. We thank the reviewer for this comment and have updated the figure.
- Figure 4 Panel b should be made clear on the figure itself that the significance thresholds shown on the venn diagrams for GPMelt and NPARC are different. We thank the reviewer for this comment and have updated the figure.
- There are no comments related to the background selection for the enrichment analysis, which is what authors justify to say their method finds appropriate novel results. If no background is selected, then the enrichment results would be expected. Did the author select the correct background (the protein identified in the experiment)? We thank the reviewer for this accurate comment, and would like to confirm that the correct background, i.e. the proteins identified in the experiment, has been selected. We added this information in the legend of Fig 4 of the main text and of S15 Fig of the supplementary: "The enrichment analysis is performed with the R package clusterProfiler [13] (v4.8.3), with background defined by the set of proteins identified in the experiment."
- Supplemental figures are barely visible at 200x zoom. Please make the legend clear to readers can easily identify what each colour on the plots represents, this is particularly relevant for S3 and S4 as its not possible to discern what is described on the text from the current figures. We thank the reviewer for this comment and have updated the figures.

We would like to thank the Guest Editor and the reviewers again for taking the time to review our manuscript and consider our responses.

References

1. Fang S, Kirk PD, Bantscheff M, Lilley KS, Crook OM. A Bayesian semi-parametric model for thermal proteome profiling. Communications biology. 2021;4(1):1–15.

2. Sridharan S, Kurzawa N, Werner T, Günthner I, Helm D, Huber W, et al. Proteome-wide solubility and thermal stability profiling reveals distinct regulatory roles for ATP. Nature communications. 2019;10(1):1–13.

3. Childs D, Bach K, Franken H, Anders S, Kurzawa N, Bantscheff M, et al. Nonparametric analysis of thermal proteome profiles reveals novel drug-binding proteins. Molecular & Cellular Proteomics. 2019;18(12):2506–2515.

4. Williams CK, Rasmussen CE. Gaussian processes for machine learning. vol. 2. MIT press Cambridge, MA; 2006.

5. Benjamini Y, Hochberg Y. Controlling the false discovery rate: a practical and powerful approach to multiple testing. Journal of the Royal statistical society: series B (Methodological). 1995;57(1):289–300.

6. Storey JD. A direct approach to false discovery rates. Journal of the Royal Statistical Society Series B: Statistical Methodology. 2002;64(3):479–498.

7. Liu Z, Barahona M. Similarity measure for sparse time course data based on Gaussian processes. In: Uncertainty in Artificial Intelligence. PMLR; 2021. p. 1332–1341.

8. Potel CM, Kurzawa N, Becher I, Typas A, Mateus A, Savitski MM. Impact of phosphorylation on thermal stability of proteins. Nature methods. 2021;18(7):757–759.

9. UniProt: the Universal Protein knowledgebase in 2023. Nucleic Acids Research. 2023;51(D1):D523–D531.

10. Gardner, Jacob R and Pleiss, Geoff and Bindel, David and Weinberger, Kilian Q and Wilson, Andrew Gordon GPyTorch: Blackbox Matrix-Matrix Gaussian Process Inference with GPU Acceleration Advances in Neural Information Processing Systems, 2018.

11. Kingma DP, Ba J. Adam: A method for stochastic optimization. arXiv preprint arXiv:14126980. 2014;.

12. R Core Team. R: A Language and Environment for Statistical Computing; 2023. Available from: `https://www.R-project.org/`.

13. Wu T, Hu E, Xu S, Chen M, Guo P, Dai Z, et al. clusterProfiler 4.0: A universal enrichment tool for interpreting omics data. The innovation. 2021;2(3).

14. Kassambara A. ggpubr: 'ggplot2' Based Publication Ready Plots; 2023. Available from: `https://CRAN.R-project.org/package=ggpubr`.
